# Determinants of eating patterns and nutrient intake among adolescent athletes: a systematic review

**DOI:** 10.1186/s12937-017-0267-0

**Published:** 2017-07-28

**Authors:** Matias Noll, Carolina Rodrigues de Mendonça, Lorena Pereira de Souza Rosa, Erika Aparecida Silveira

**Affiliations:** 1 Instituto Federal Goiano – Campus Ceres, Rodovia GO-154 - Km 3, Ceres, Goiás, GO 76300-000 Brazil; 2 Postgraduate Program in Health Sciences, Faculty of Medicine, Universidade Federal de Goiás, Goiás, Brazil

**Keywords:** Youth, Sport, Nutrition, Eating behavior, Dietary intake, food intake

## Abstract

**Background:**

This review aims to update the influences of sport modalities, sport performance, and non-exercise-related determinant, on eating patterns and nutrient intake outcomes among adolescent competitive athletes.

**Methods:**

The PubMed and Scopus databases were searched for the last 20 years. Observational and intervention studies of all languages on eating patterns and nutrient intake in adolescent (10- to 19-year-old) competitive athletes were included. Study quality and risk of bias were assessed using a Downs and Black instrument. Moreover, the Grading of Recommendations, Assessment, Development and Evaluations (GRADE) evidence system was used to assess the strength of the body of evidence.

**Results:**

Regarding outcomes of 21 included articles, 95.2% of studies focused on nutrient analysis, whereas few of the included articles reported eating patterns. As determinants, most studies analyzed the influences of sport-related (*n* = 10) and demographic factors (*n* = 8), among which only sport modalities were significantly associated with nutrient intake. Age and sex were not significantly associated with nutrient intake. All studies were observational, and most were cross-sectional (*n* = 17) and conducted in developed countries such as the United States and European nations. Most studies evaluated boys, and the sport that received the most attention was soccer.

**Conclusions:**

Athletes do not adjust their nutrient intake or food choice to the demands of the training load or different training sessions, while sport modalities significantly influenced nutrient intake. Moreover, results concerning demographic determinants were inconsistent and thus remain inconclusive.

**Trial registration:**

Prospero CRD42016043310.

**Electronic supplementary material:**

The online version of this article (doi:10.1186/s12937-017-0267-0) contains supplementary material, which is available to authorized users.

## Background

The benefits and drawbacks of sports for adolescents have been demonstrated [1–4]. Advantages include substantial evidence of psychological and social benefits [5, 6], academic benefits [2], and health benefits [7]. Participation in some sports is associated with iron-deficient anemia [8], high dietary supplement intake [9–11], and sudden death among athletes [12, 13]. Therefore, researchers have raised concerns about the diet quality of adolescent athletes [14–18].

However, few studies have investigated eating patterns and nutrient intake [19, 20] in adolescent athletes [18, 21, 22]. Consequently, there is no consensus as to whether participation in sports is associated with improved eating patterns – i.e. food choice and frequency – or macro- and micronutrient intake [23, 24]. Here, we aimed to systematically review the determinants of eating patterns and nutrient intake among adolescent competitive athletes.

The present review is the first to target adolescent athletes and focused on the following research questions: a) whether sports characteristics (i.e. sport modality and training aspects) are associated with eating patterns and nutrient intake and b) whether demographic, socioeconomic, environmental, psychosocial, and cultural factors are determinants of eating patterns and nutrient intake. We further aimed to identify gaps in the literature in this field and priority areas for future research. Such information may be valuable for promoting healthy habits that benefit athletes throughout their lives.

## Methods

### Protocol and registration

This systematic review was registered with the International Prospective Register of Systematic Reviews (PROSPERO) (protocol number: CRD42016043310) [25] and conducted according to the PRISMA (Preferred Reporting Items for Systematic Reviews and Meta-analyses) guidelines [26] for the identification, screening, eligibility, and inclusion of articles.

### Search strategy and eligibility criteria

In August 2016, two independent researchers searched the PubMed and Scopus databases, with no language restriction, for articles published between January 1996 and August 2016. The detailed search strategy is presented as Additional file 1.

We included articles with the following characteristics: (a) a research population consisting of adolescent (10−19 years old, as defined by the World Health Organization [27]) competitive sports athletes, (b) assessment of eating patterns and/or nutrient intake outcomes and their determinants, and (c) observational or interventional studies. In this context, competitive sport was defined as “a human activity capable of achieving a result requiring physical exertion and/or physical skill which, by its nature and organization, is competitive and is generally accepted as being a sport” [5, 28]. Studies that addressed ‘exercise,’ ‘physical activity,’ ‘physical education,’ or ‘recreation’ were not included [5]. Eating patterns were understood as food choices and the frequency of meals and foods; and nutrient intake was defined as macro- and micronutrient intakes and energy intake [16, 29, 30].

The exclusion criteria were: (a) evaluation of eating disorders; (b) evaluation of supplement intake; (c) studies with incomplete data or review articles; (d) populations that included pregnant and lactating women, hospitalized adolescents, disabled people, or amputees; (e) mixing of athletes with non-athletes, unless the athletes’ data were reported separately or could be calculated from the data provided; and (f) mixing of adolescents (10−19 years old) with other ages, unless the adolescents’ data were reported separately or could be calculated from the data provided.

### Review process

**Fig. 1:**
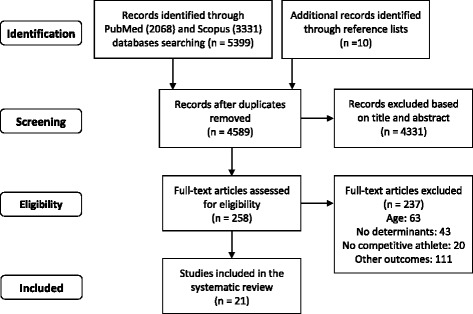
Flow chart of the data search: systematic review

After executing the search strategy (Fig. 1), duplicate articles were removed. Two reviewers (MN and CRM) then independently screened the titles and abstracts of all articles that were identified in the literature search for inclusion in the systematic review. Disagreement on manuscript inclusion was assessed by concordance analysis (percentage of agreement and the kappa test) and resolved by a third reviewer (LPSR) [31]. The remaining articles were read in full and evaluated to determine their eligibility based on the inclusion and exclusion criteria. Finally, the eligible articles were included in the present systematic review. In addition, the reference lists of included articles were searched to identify additional studies missed by database searches.

### Data extraction, quality assessment, and synthesis

From the selected articles, the following data were extracted: authors, publication year, sample size, sex, age, location, sport modality, study design, presence of a nutritionist in the team of health professionals, outcome, instrument, determinants, and major findings. Study quality and risk of bias were assessed using a 27-item Downs and Black instrument [32]. For observational studies, not all items on the Downs and Black checklist were generally applicable, and a subset of ten questions (Questions 1−3, 6, 7, 10−12, 18, and 20) was used [33]. A summary quality score for each manuscript was calculated by expressing the number of compliant items as a percentage of the total. Furthermore, the Grading of Recommendations, Assessment, Development and Evaluations (GRADE) evidence system was used to assess the strength of the body of evidence [34]. For each research outcome, quality of evidence was ultimately given one of four grades: high quality, moderate quality, low quality, or very low quality [35].

We also analyzed whether the authors of included studies addressed the impact of possible conflicts of interest and information on ethical approval [36]. Data were extracted and assessed by two independent reviewers (MN and CRM), and disagreements were resolved by a third reviewer (LPSR). When relevant data were not available in the manuscript, a researcher (MN) contacted the authors directly to fill these gaps.

## Results

### Literature search and study selection

A total of 5399 articles were identified through the PubMed and Scopus database searches, and 10 additional records were identified through reference lists. After removal of duplicates, the titles and abstracts of 4589 records were screened. High concordance (94.7% concordance; adjusted kappa = 0.89; 95% CI: 0.88−0.90) [37] between reviewers was found, and 258 articles were selected for full text review (Fig. 1).

After the full text analysis, 21 articles met the eligibility criteria and were included in the present systematic review. Among the 237 excluded studies, the most common reason for exclusion was that they evaluated other outcomes (*n* = 111), followed by not assessing adolescents, not evaluating the determinants of eating patterns and/or nutrient intake, and not evaluating competitive athletes (Fig. 1).

### Study characteristics

All studies were observational in design, and 80.9% were cross-sectional [38–54] (see Additional file 2). Sample size ranged from 9 to 1138 athletes, with the majority of studies (85.7%) including less than 100. Most studies evaluated boys [40, 42, 44–46, 49, 50, 52, 55, 56]. Further, 80.9% studies were conducted in developed countries such as the United States [47, 48, 57] and European nations [38–43, 45, 46, 53, 56, 58, 59]. The sport that received the most attention was soccer [40, 42, 44, 45, 52, 55, 56]. Only two studies [41, 56] reported that the athletes received practical and individual recommendations from a nutritionist.

### Outcomes and determinants

The characteristics of the included studies are shown in Table 1. First, the most common method of evaluation (80.9%) was the analysis of food records ranging from 1 to 7 days. From these records, 15 studies evaluated three or more days, one study [44] evaluated 2 days, and another study [51] evaluated just 1 day food records. Second, one study used a food weight method with 1 day [46] and three studies used a food weight method with 5 days or more [39, 42, 52]. Third, four studies used a validated food frequency questionnaire [42, 43, 51] and one [41] used a questionnaire specifically validated for that study. Fourth, one study used a food diary associated with food records [54].

**Table 1:** The design of human trials for estimating food patterns and nutrient intakes among adolescent athletes distributed by sport and country

Sport	Country	Sample size	Sex	Age	Study design	Outcome: dietary assessment	Evaluated determinants
Athletics	Belgium [58]	60	M: 51.7%	12−18	Longitudinal (3-year follow-up)	NU/FO: 7-day FR	Age, Sex
Cycling Running	China [49]	24	M: 100%	Cyclists: 15.3 ± 0.7 Runners: 15.5 ± 1.3	Cross-sectional	NU: 7-day FR	Sport modality
Judo	France [38]	9	F: 100%	15−16	Cross-sectional (3 and 1 week before competition)	NU: 7-day FR	Training (time)
Judo	Tunisia [50]	15	M: 100%	18 ± 1	Cross-sectional (before, during, and after Ramadan)	NU: 3-day FR	Ramadan
Pentathlon	Brazil [51]	56	M: 60.7%	10−18	Cross-sectional	NU: 1-day FR, FFQ	Sex
Rugby	France [46]	14	M: 100%	15−16	Cross-sectional (control session, rugby session and exercise session)	NU: 1-day weighed food	Training (session)
Several sports	Brazil [54]	326	M: 62.6%	11−14	Cross-sectional	NU: 3-day FR, Food diary	Sport modality
Several sports	Germany [41]	1138	M: 56.1%	14−18	Cross-sectional	FO: Questionnaire	Sex, Sport modality, School, Place to eat, Nutrition
Skating	US [47]	28	M: 42.9%	M: 16 ± 1.5 F: 14 ± 1.6	Cross-sectional	NU: 4-day FR	Sex
Skating	US [48]	94	M: 48.9%	M: 17.2 ± 3.0 F: 15.0 ± 2.4	Cross-sectional	NU: 3-day FR	Meals
Skating	US [57]	18	F:100%	14−16	Longitudinal (9 months, pre-, competitive, and off-season)	NU: 3-day FR	Training (time)
Soccer	France [56]	180	M: 100%	13−16	Longitudinal (3-year follow-up)	NU: 5-day FR	Age
Soccer	Israel [44]	19	M: 100%	14−16	Cross-sectional (before and during Ramadan)	NU: 2-day FR	Ramadan
Soccer	Italy [40]	43	M: 100%	15−17	Cross-sectional (two evaluations 3 months apart)	NU: 4-day FR	Training (time)
Soccer	Spain [42]	22	M:100%	14−16	Cross-sectional	NU: 6-day weighed food FO: FFQ	Food preference
Soccer	Spain [45]	57	M: 100%	Team A: 14 ± 0.3 B: 15 ± 0.2 C: 16.6 ± 0.6	Cross-sectional	NU: 3-day FR	Age
Soccer	Spain [52]	62	M: 100%	13−19	Cross-sectional	NU: 5-day weighed food	Menu settings
Soccer	UK [39]	10	M: 100%	15.4 ± 0.3	Cross-sectional (4 training days)	NU: 7-day FR, NU: 7-day weighed food	Training (session)
Swimming	Greece [59]	9	M: 45.5%	M: 18.4 ± 1.2 F: 17.3 ± 1.7	Longitudinal (4 evaluations in 8 months)	NU: 3-day FR	Sex Training (time)
Swimming	Spain [43]	36	M: 61.1%	M: 15.5 ± 0.4 F: 14.6 ± 0.4	Cross-sectional	NU: 3-day FR FO: FFQ	Sex
Volleyball	Greece [53]	65	F: 100%	14−19	Cross-sectional	NU: 3-day FR	Training (categories)

*NU* nutrient intake, *FO* food intake, *FR* food record, *FFQ* food frequency questionnaire, *US* United States, *UK* United Kingdom

The 21 articles were analyzed and categorized based on their assessment of two broad outcomes: food and nutrient intake. Only four studies presented results regarding food intake [41–43, 58]. As determinants for these outcomes, most studies analyzed the influences of sport-related factors (*n* = 10), age (*n* = 3), sex (*n* = 6), and Ramadan (*n* = 2). Other rarely evaluated determinants included food preference, menu settings, meal patterns, place of eating, and type of school (Table 1). The determinants to both outcomes are described below:

- Sports training and modality

Ten studies evaluated the influence of sport-related factors on food and nutrient intake. Five studies found that athletes did not adjust their nutrient intake to the demands of different training loads and sessions. Briggs et al. [39] compared four training days (heavy and moderate training, match, and rest) and found no differences in macronutrient intake. Caccialanza et al. [40] performed two evaluations separated by a 3-month period and also found no significant variations in reported energy, macronutrient, fiber, or cholesterol intakes between evaluations. Papadopoulou et al. [53] compared two training categories of volleyball players and found no differences in macro- or micronutrient intakes with the exception of fat ingestion. Kabasakalis et al. [59] and Ziegler et al. [57] reported no significant differences in macronutrient intakes in 8- and 9-month studies, respectively. Similarly, Boisseau et al. [38] found no differences in micro- and macronutrient intake across weeks of a training period with the exception of lipid ingestion and carbohydrate. However, Thivel et al. [46] observed differences in meal composition in a study comparing different training sessions (control session, rugby session, and exercise session) (Table 2).

**Table 2:** The sport dependent food and nutrient intakes classified according to sport modality and country

Sport	Country	Categories	Criteria of statistical comparison									
			Foods	Sweets	EI	Prot	CHO	Fat	Chol	Fiber	Vit	Min
Athletics	Belgium [58]	Age	= Pas, Po, R, WhiBr, Fr	N/A	N/A	=	=	N/A	N/A	N/A	N/A	N/A
		B vs G	↑ Pas, Po, R, WhoBr, So = Fr, Veg	N/A	↑	=	↑	N/A	N/A	↑	N/A	N/A
Cycling Running	China [49]	Cyclists vs runners	N/A	N/A	↑	↑	↑	↑	↑	=	= A, C	↑Fe, Mg, Mn, Se, Zn
Judo	France [38]	Training: week 3 vs 1	N/A	N/A	↑	=	↑	↑	↑	=	=	=
Judo	Tunisia [50]	Ramadan: Bef vs Dur vs After	N/A	N/A	=	=	=	=	N/A	N/A	N/A	N/A
Pentathlon	Brazil [51]	B vs G	N/A	N/A	=	=	=	=	N/A	N/A	N/A	N/A
Rugby	France [46]	Lu: Exerc vs Rugby vs Con	N/A	N/A	=	=	=	↑ Exerc	N/A	N/A	N/A	N/A
		Sn: Exerc vs Rugby vs Con	N/A	N/A	=	↑ Con	↑ Exerc	↑ Rugby	N/A	N/A	N/A	N/A
		Di: Exerc vs Rugby vs Con	N/A	N/A	↑ Exerc	↑ Rugby	=	=	N/A	N/A	N/A	N/A
Several sports	Brazil [54]	Endur sports vs other sports	N/A	N/A	=	↑	=	=	N/A	N/A	N/A	N/A
Several sports	Germany [41]	B vs G	↓ Vit fib foods ↑ An prod	N/A	N/A	N/A	N/A	N/A	N/A	N/A	N/A	N/A
		Endur vs others	↑ Vit fib foods	N/A	N/A	N/A	N/A	N/A	N/A	N/A	N/A	N/A
		Nutrition plan	↑ Vit fib foods	N/A	N/A	N/A	N/A	N/A	N/A	N/A	N/A	N/A
		On a diet	N/A	↓	N/A	N/A	N/A	N/A	N/A	N/A	N/A	N/A
		Boarding student	↑ An prod	N/A	N/A	N/A	N/A	N/A	N/A	N/A	N/A	N/A
		Snack bar	N/A	↑	N/A	N/A	N/A	N/A	N/A	N/A	N/A	N/A
Skating	US [47]	B vs G	N/A	=	↑	↑	↑	↑	↑	N/A	↑B1, B3 = A, B2, B6, B9, B12, C, D, E,	↑Fe, P, Na, Zn = Ca, K, Mg, I
Skating	US [48]	B: Bkf vs Lu vs Di vs Sn	N/A	N/A	↑ Di	↑ Di	↑ Di	↑ Lu	↑ Bkf	↑ Di	↑ Bkf: B9	↑Di: Fe, Ca
		G: Bkf vs Lu vs Di vs Sn	N/A	N/A	↑ Di	↑ Di	=	↑ Lu	↑ Di	↑ Di	↑ Bkf: B9	↑Di: Ca ↑ Bkf: Fe
Skating	US [57]	Pre- vs Comp vs Off-seasons	N/A	N/A	=	=	=	=	=	=	↑ Pre: B1, B2, B3, B6, B9, C, D, E	↑ Pre: Zn = Fe, Na, I, Mg, P, Ca, K
Soccer	France [56]	Age	N/A	N/A	↑	↑	↑	N/A	N/A	N/A	N/A	↑ Ca, FE
Soccer	Israel [44]	Ramadan: Bef vs Dur	N/A	N/A	=	=	=	=	N/A	N/A	N/A	N/A
Soccer	Italy [40]	Training	N/A	N/A	=	=	=	=	=	=	N/A	N/A
Soccer	Spain [42]	Food preference	=	N/A	=	=	=	=	=	=	N/A	N/A
Soccer	Spain [45]	Age	N/A	=	↓	=	↓	=	N/A	=	N/A	N/A
Soccer	Spain [52]	Fixed menu vs Buffet-style	N/A	N/A	↑	=	↑	=	=	N/A	↑D, E = A	↑Mg = Ca
Soccer	UK [39]	Match vs Heavy vs Mod vs Rest	N/A	N/A	N/A	=	=	=	N/A	N/A	N/A	N/A
Swimming	Greece [59]	B vs G	N/A	N/A	=	N/A	N/A	N/A	↑	=	N/A	N/A
		Training	N/A	N/A	=	=	=	=	N/A	N/A	N/A	N/A
Swimming	Spain [43]	B vs G	↓ Fr, Nuts = Veg, Leg, Fish, Meat, Eggs, Cereals, DP	N/A	↑	↑	=	=	=	N/A	↑ B1, B2, B3, B6, E = A, C, D, B12	↑ Ca, Mg, P, Fe, K, Na = Zn
Volleyball	Greece [53]	Natl vs Champ players	N/A	N/A	↑	=	=	↑	=	=	= A, B, C	= Ca, Fe, Mg, Zn

Abbreviations (alphabetical order):

*An prod* Animal products, *B* Boys, *G* Girls, *Bef* Before, *Bkf* breakfast, *Champ* Championship, *CHO* Carbohydrate, *Chol* Cholesterol, *Comp* Competitive season, *Con* Control, *Di* Dinner, *DP* Dairy products, *Dur* During, *EI* Energy intake, *Endur* Endurance, *Exerc* Exercise, *Fr* Fruits, *Leg* Legumes, *Lu* Lunch, *Min* Mineral, *Mod* Moderate, *Natl* National, *Pas* Pasta, *Po* Potato, *Pre* Pre-season, *Prot* Protein, *R* Rice, *Sn* Snacks, *So* Soup, *Veg* Vegetables, *Vit fib foods* Vitamin and fiber-rich foods, *Vit* Vitamin, *WhiBr* White bread, *WhoBr* Wholegrain bread

= means no significant differences; ↑ means statistical significant increase; ↓ means statistical significant decrease; ^N/A^ means not available

The details of each study are presented in Table 1

Three studies verified the association between sport modalities and food and nutrient intake. Diehl et al. [41] found that endurance sport athletes eat more foods high in vitamins and fiber than do competitors in strength sports, ball games, or aesthetic sports. Sousa et al. [54] also evaluated several sports and found differences in protein intake. Tong et al. [49] evaluated runners and cyclists and found that intakes of macronutrients as well as micronutrients were higher in cyclists.

b) Demographic determinants

Conflicting results were reported concerning demographic (sex and age) determinants. Diehl et al. [41] found better food intake in female athletes, such as consumption of more products high in vitamins and fiber and fewer animal products. Martínez et al. [43] also found higher fruit intake among female athletes. However, Aerenhouts et al. [58] found no sex differences in fruit and vegetable consumption, although boys ate more wholegrain bread, pasta, rice, and potatoes than girls. Regarding nutrient intake, Ziegler et al. [47] demonstrated that male athletes had higher total energy and macro- and micronutrient intakes than girls did, and Kabasakalis et al. [59] found higher cholesterol intakes among males. Similar results were found by Martínez et al. [43], who observed higher intakes of macro- and micronutrients among males athletes. By contrast, Coutinho et al. [51], found no differences in energy or nutrient intake between the sexes. In short, the reported role of sex as a determinant for both food and nutrient intake outcomes is inconsistent. These conflicting results could be due to the different methods used; for example, Coutinho et al. [51] evaluated just one-day food records (Table 2).

Regarding age influences, Ruiz et al. [45] evaluated three different age groups and carbohydrate intake was found to decrease with increasing age, in parallel with a decrease in the percentage of total energy ingested at breakfast, morning break, and afternoon break. Leblanc et al. [56] found that energy, macro-, and micronutrient intakes increased significantly over a 3-year follow-up period. In another 3-year follow-up study, Aerenhouts et al. [58] demonstrated no differences between ages with regard to nutrient intake and eating patterns. Therefore, results concerning the role of age as a determinant for both food and nutrient intake outcomes are also inconclusive.

c) Others determinants

The relation between Ramadan and nutrient intake were investigated by two studies [44, 50]; no significant changes on macro- and micronutrient intake before, during, and after Ramadan were found. Other determinants, although evaluated by single studies, were also found to be important. Ziegler et al. [48] examined the contribution of meal patterns (breakfast, lunch, dinner, and snack) on micro- and macronutrient and found differences between the predominant sources of fat, cholesterol, and dietary fiber intake at lunch and dinner for girls and boys. Garrido et al. [52] evaluated menu settings and found that fixed ‘menu-style’ menus have more total energy, cholesterol, and micronutrient intakes than do flexible ‘buffet-style’ menus. One study [42] demonstrated that food preference does not interfere with nutrient intake or the daily number of food portions consumed. Regarding eating patterns, Diehl et al. [41] reported several results: athletes who had a nutrition plan and information from a nutritionist ate more products high in vitamins and fiber; eating meals at the school cafeteria was associated with increased consumption of animal products; and eating at a snack bar and on the way were associated with increased consumption of sweets and snacks (Table 2).

### Data quality assessment and strength of evidence

The Downs and Black checklist scores ranged from 60% to 100% (see Additional file 3). Principal risks of bias arose from the lack of reporting of probability values, targeted samples, and recruitment of a representative population. Only two studies [41, 54] evaluated a nationally representative sample. Strength of evidence classification using the GRADE methodology indicated that 12 studies were of very low quality. Only the studies by Diehl et al. [41] and Sousa et al. [54] presented a moderate strength of evidence. Notably, just one-fourth of studies [39, 41, 46, 51, 54] clearly stated that there were no conflicts of interest, and most studies (*n* = 13) reported ethical approval (see Additional file 3).

## Discussion

Our study is the first to systematically review the determinants of eating patterns and nutrient intake among adolescent competitive athletes. Regarding outcomes, most studies focused on nutrient analysis, and few of the included articles reported results concerning eating patterns. Our findings suggest that athletes did not adjust their nutrient intake to the demands of the training load and different training sessions. However, sport modalities significantly influenced their nutrient intake and eating patterns. Such evidence is valuable to improve diet quality that benefits athletes throughout their lives. Moreover, demographic determinants (age and sex) demonstrated conflicting results, and the cultural determinant of Ramadan did not influence nutrient intake.

We were unable to establish a strong relationship between eating patterns or nutrient intake and environmental (fixed menu [52], eating in a cafeteria or snack bar [41]) or psychosocial determinants (food preference [42]; meal patterns [48], information from a nutritionist, nutritional plan, maintenance on a diet [41]) because these variables were each evaluated in only one study. Moreover, we did not identify any studies that evaluated socioeconomic characteristics as possible determinants of eating patterns or nutrient intake.

Several reviews focused on other populations have identified many factors that can influence diet quality. In a nonsystematic review, Birkenhead and Slater [23] highlighted the multidimensional nature of food choices and indicated that nutritional knowledge and physiological, social, and economic factors were associated with food choices. Scaglioni et al. [60] described how eating patterns involve a complex interaction of genetic, familial, and environmental factors. Furthermore, studies focused on non-athlete students [61–63] identified others important determinants including parental control, cultural traditions, climatic factors, and smoking and drinking. Thus, compared to youth [64] and adults [65], there is a lack of evidence concerning the evaluation of determinants for adolescent athletes.

Regarding the two broad outcomes, few studies reported on eating patterns [41–43, 58]. Until recently, the relationship between eating patterns and health was underestimated, and most guidelines treated foods as mere nutrient carriers [22, 66, 67]. However, current investigations have extended beyond the simple intake of nutrients to consider food choice as well as the level of processing to which foods are subjected [68, 69]. Data concerning eating patterns are essential [20, 70], because nutrition-specific education programs may impact training and performance and should target not only nutrients, but also both food choice and eating habits [22, 48]. Erdman et al. [71] and Iglesias et al. [21] suggested that research on eating patterns and food sources is indispensable because it may improve athletes’ knowledge, which is essential to effectively advise them with tangible data [4].

The present systematic review revealed several notable gaps in the literature in this field. First, no study investigated athletes from South Africa, Central America, or Oceania, and most were conducted in developed countries. Second, male athletes were the focus of more studies. Third, most studies were cross-sectional and evaluated small samples. Fourth, soccer was the most commonly investigated sport, while other sports received less attention. Fifth, due the wide range of aims, measurement tools, outcomes, and determinants assessed, a meta-analysis cannot be carried out. Sixth, food records and food weight protocols including less than 3 days are not representative to calculate nutrient intake. Furthermore, other possibly important determinants were not investigated, such as family educational and socioeconomic level, parental rules, coach’s knowledge of nutrition and rules, athlete’s nutritional knowledge, sponsorship and commercial activities, presence of a nutritionist in the sport team, smoking and drinking, sedentary patterns, and urban or rural residence. These other possible determinants should be considered in future studies [70, 72, 73].

The strength of evidence was low, and the analysis of the methodological quality of the studies indicated that the principal risk of bias was that the sample was not representative of the population. However, convenience samples are common in epidemiological studies [74]. Moreover, we observed that most articles did not contain a clear statement regarding conflict of interest, and this consideration should be taken into account in future studies. This issue has been cited as a concern of the Pan American Health Organization, which has warned of the dangers of conflicts of interest related to nutrition [75]. The World Health Organization addressed this issue in 2015 [76].

Overall, based on the gap in knowledge on eating patterns, the limited number of determinants analyzed, and the results regarding risk of bias and quality of evidence, our results highlight some interesting opportunities for future research, particularly the performance of longitudinal and experimental studies; greater research efforts in developing countries; evaluation of representative samples, preferably including both sexes; assessment of a wide variety of sports, preferably within the same study to allow for comparisons between them; and inclusion of a wider range of determinants. The greater promotion of adequate nutrition during adolescence may optimize training performance as well as improve nutritional knowledge and healthy habits that may benefit the athletes beyond the end of their sports careers [22, 24, 41, 77]. Clubs and sporting organizations may provide an ideal outlet to introduce health-related policies, and preferably with a nutritionist in the team of health professionals [78]. New studies may contribute to the development of preventive programs and health strategies and policies aimed at adolescent athletes [4, 79, 80] as well as further promote greater collaboration between researchers and research groups through multi-center studies.

Strengths of this study include the identification of specific determinants and the focus on an existing gap in knowledge related to the evaluation of eating patterns among adolescent competitive athletes. Furthermore, this review has several methodological strengths, including the absence of a language restriction; high concordance between reviewers in title and abstract screening; inclusion of study quality and risk of bias analysis; and analyses of conflicts of interest and ethical approval, which are often omitted from reviews.

## Conclusion

In summary, athletes do not adjust their nutrient intake to the demands of the training load or different training sessions, while sport modalities significantly influenced nutrient intake. Moreover, demographic determinants are inconsistent and remain inconclusive. Lastly, nutrient intake has received more attention than eating patterns in studies of adolescent athletes. Furthermore, future research should be developed to improve the quality of evidence regarding determinants of eating patterns and nutrient intake.

## Additional files

Additional file 1: Search strategy. The PubMed search terms were: (((((((((((((((((food choice[Title/Abstract]) OR food intake[Title/Abstract]) OR food consumption[Title/Abstract]) OR eating behavior[MeSH Terms]) OR nutrition assessment[MeSH Terms]) OR food preference[MeSH Terms]) OR health behavior[MeSH Terms]) OR food habits[MeSH Terms]) OR diet, food, and nutrition[MeSH Terms]) OR nutritional status[MeSH Terms]) OR feeding behavior[MeSH Terms]) OR eating[MeSH Terms]) OR food and beverages[MeSH Terms]) OR diet[MeSH Terms]) OR food[MeSH Terms]) AND (((adolescent[MeSH Terms]) OR students[MeSH Terms]) OR minors[MeSH Terms])) AND ((athletes[MeSH Terms]) OR sports[MeSH Terms]). This search strategy was adapted for the Scopus database. (DOCX 10 kb)

Additional file 2: Quantitative characteristics of the 21 articles included in the systematic review. (DOCX 13 kb)

Additional file 3: Methodological quality assessment and strength of evidence. (DOCX 2818 kb)

## Abbreviations

GRADE: Grading of recommendations, assessment, development and evaluations
PRISMA: Preferred reporting items for systematic reviews and meta-analyses
PROSPERO: International Prospective Register of Systematic Reviews
