# Supplementary material for: Determinants of eating patterns and nutrient intake among adolescent athletes: a systematic review
Source: Nutr J. 2017 Jul 28;16:46. doi: 10.1186/s12937-017-0267-0 (PMC5534032; doi:10.1186/s12937-017-0267-0)
Supplement: Supplementary file 1 — Search strategy. The PubMed search terms were: (((((((((((((((((food choice[Title/Abstract]) OR food intake[Title/Abstract]) OR food consumption[Title/Abstract]) OR eating behavior[MeSH Terms]) OR nutrition assessment[MeSH Terms]) OR food preference[MeSH Terms]) OR health behavior[MeSH Terms]) OR food habits[MeSH Terms]) OR diet, food, and nutrition[MeSH Terms]) OR nutritional status[MeSH Terms]) OR feeding behavior[MeSH Terms]) OR eating[MeSH Terms]) OR food and beverages[MeSH Terms]) OR diet[MeSH Terms]) OR food[MeSH Terms]) AND (((adolescent[MeSH Terms]) OR students[MeSH Terms]) OR minors[MeSH Terms])) AND ((athletes[MeSH Terms]) OR sports[MeSH Terms]). This search strategy was adapted for the Scopus database. (DOCX 10 kb) [file 12937_2017_267_MOESM1_ESM.docx]

Additional file 1. Search strategy.

| The PubMed search terms were: (((((((((((((((((food choice[Title/Abstract]) OR food intake[Title/Abstract]) OR food consumption[Title/Abstract]) OR eating behavior[MeSH Terms]) OR nutrition assessment[MeSH Terms]) OR food preference[MeSH Terms]) OR health behavior[MeSH Terms]) OR food habits[MeSH Terms]) OR diet, food, and nutrition[MeSH Terms]) OR nutritional status[MeSH Terms]) OR feeding behavior[MeSH Terms]) OR eating[MeSH Terms]) OR food and beverages[MeSH Terms]) OR diet[MeSH Terms]) OR food[MeSH Terms]) AND (((adolescent[MeSH Terms]) OR students[MeSH Terms]) OR minors[MeSH Terms])) AND ((athletes[MeSH Terms]) OR sports[MeSH Terms]). This search strategy was adapted for the Scopus database. |
| --- |
