# Supplementary material for: Determinants of eating patterns and nutrient intake among adolescent athletes: a systematic review
Source: Nutr J. 2017 Jul 28;16:46. doi: 10.1186/s12937-017-0267-0 (PMC5534032; doi:10.1186/s12937-017-0267-0)
Supplement: Supplementary file 2 — Quantitative characteristics of the 21 articles included in the systematic review. (DOCX 13 kb) [file 12937_2017_267_MOESM2_ESM.docx]

Additional file 2 Quantitative characteristics of the 21 articles included in the systematic review.

| Characteristics |  | Number of studies (%) |
| --- | --- | --- |
| Publication Year | 1996−2002 | 5 (23.8) |
|  | 2003−2009 | 9 (42.8) |
|  | 2010−2016 | 7 (33.4) |
| Study Design | Cross-sectional | 17 (80.9) |
|  | Longitudinal | 4 (19.1) |
|  | Intervention | 0 (0) |
| Region/Country | European countries | 13 (61.8) |
|  | United States | 3 (14.3) |
|  | Brazil | 2 (9.5) |
|  | Israel | 1 (4.8) |
|  | China | 1 (4.8) |
|  | Tunisia | 1 (4.8) |
| Sex | Male sex only | 10 (47.6) |
|  | Female sex only | 3 (14.3) |
|  | Mixed | 8 (38.1) |
| Sample size | <100 | 18 (85.7) |
|  | 100–1000 | 2 (9.5) |
|  | >1000 | 1 (4.8) |
| Sport | Soccer | 7 (33.3) |
|  | Skating | 3 (14.3) |
|  | Others | 11 (52.4) |
| Outcome | Food intake | 1 (4.8) |
|  | Nutrient intake | 17 (80.9) |
|  | Both | 3 (14.3) |
