# Supplementary material for: Determinants of eating patterns and nutrient intake among adolescent athletes: a systematic review
Source: Nutr J. 2017 Jul 28;16:46. doi: 10.1186/s12937-017-0267-0 (PMC5534032; doi:10.1186/s12937-017-0267-0)
Supplement: Supplementary file 3 — Methodological quality assessment and strength of evidence. (DOCX 2818 kb) [file 12937_2017_267_MOESM3_ESM.docx]

Additional file 3 Methodological quality assessment and strength of evidence.

| Study | Conflict  of interest | Ethical approval |  | Downs and Black checklist | | | | | | | | | | |  | GRADE |
| --- | --- | --- | --- | --- | --- | --- | --- | --- | --- | --- | --- | --- | --- | --- | --- | --- |
|  |  |  |  | 1 | 2 | 3 | 4 | 5 | 6 | 7 | 8 | 9 | 10 | Score |  |  |
| Aerenhouts (2011) | * | Yes |  | 1 | 1 | 1 | 1 | 1 | 1 | 0 | 0 | 1 | 1 | 80% |  | 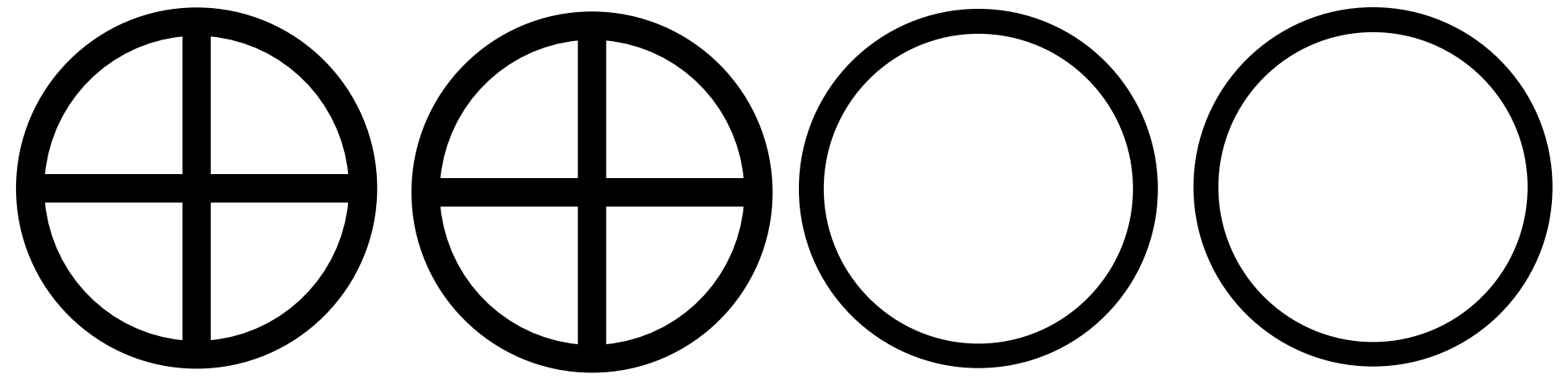 |
| Boisseau (2005) | * | Yes |  | 1 | 1 | 1 | 1 | 1 | 0 | 0 | 0 | 1 | 1 | 70% |  | 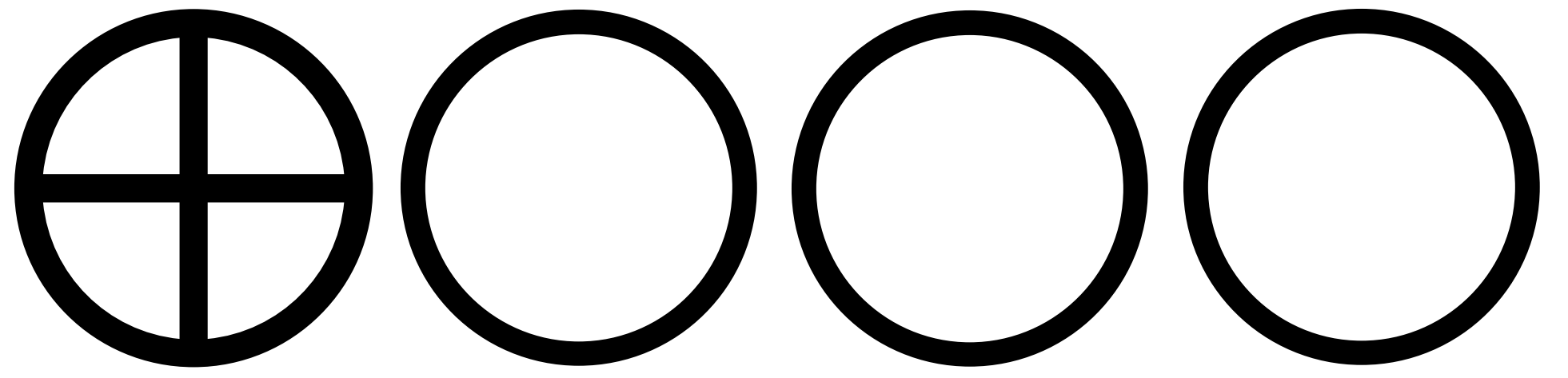 |
| Briggs (2015) | No | Yes |  | 1 | 1 | 1 | 1 | 1 | 1 | 0 | 0 | 1 | 1 | 80% |  | 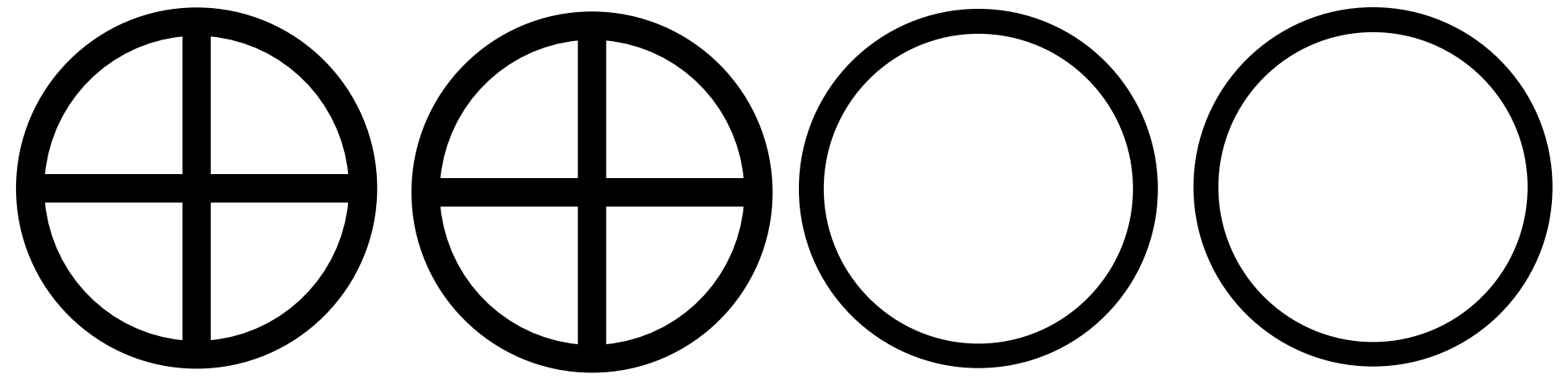 |
| Caccialanza (2007) | * | * |  | 1 | 1 | 1 | 1 | 1 | 0 | 0 | 0 | 1 | 1 | 70% |  | 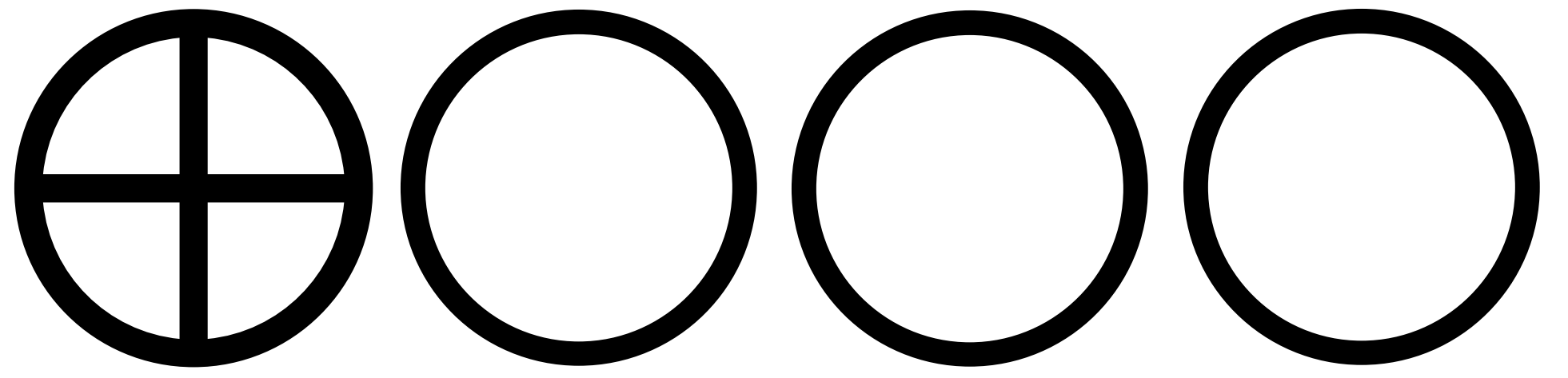 |
| Chaouachi (2008) | * | Yes |  | 1 | 1 | 1 | 1 | 1 | 0 | 0 | 0 | 1 | 1 | 70% |  | 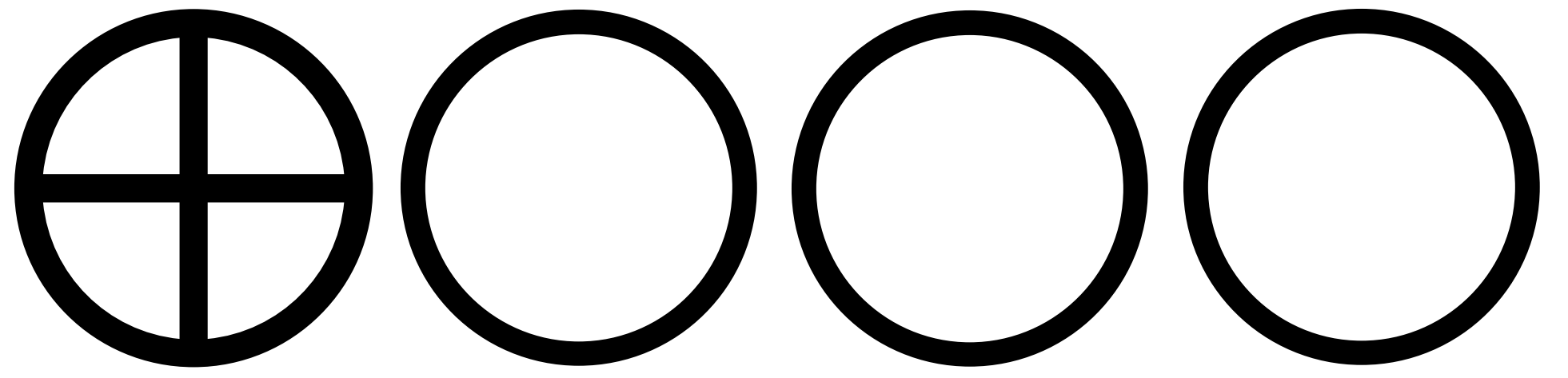 |
| Coutinho (2016) | No | Yes |  | 1 | 1 | 1 | 1 | 1 | 0 | 0 | 0 | 1 | 1 | 70% |  | 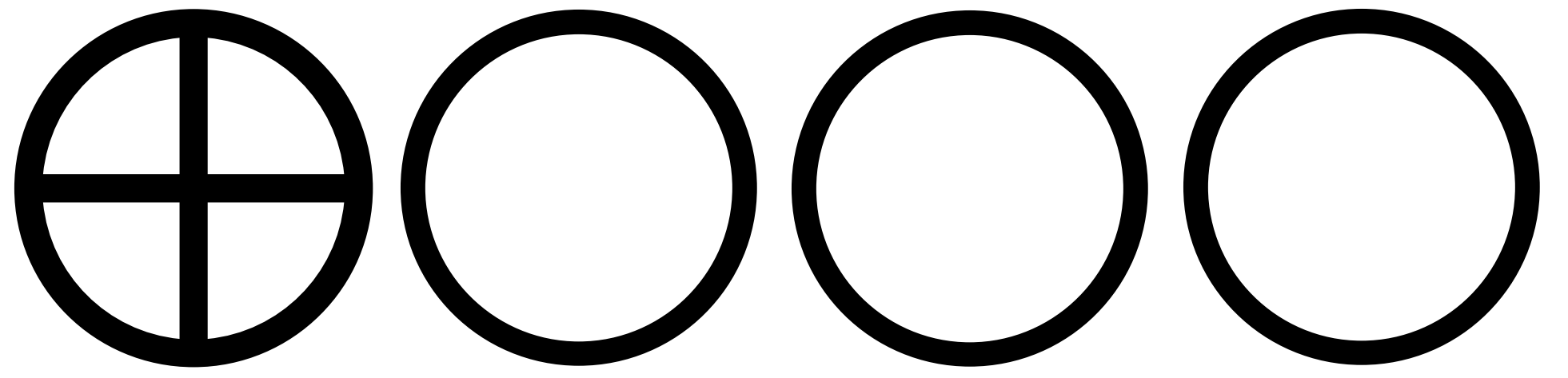 |
| Diehl (2013) | No | Yes |  | 1 | 1 | 1 | 1 | 1 | 0 | 1 | 1 | 1 | 1 | 90% |  | 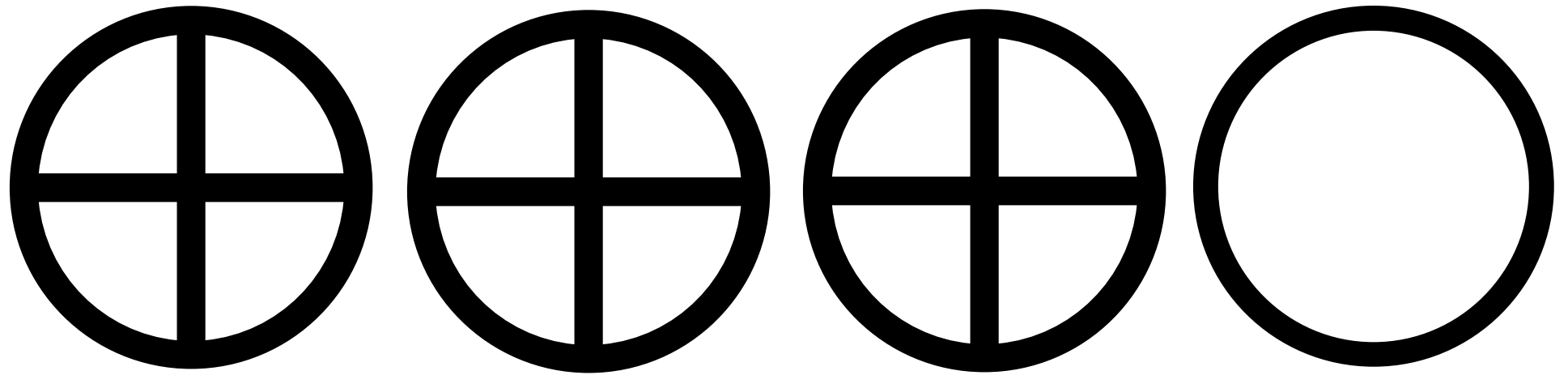 |
| Garrido (2007) | * | * |  | 1 | 1 | 1 | 1 | 1 | 0 | 0 | 0 | 1 | 1 | 70% |  | 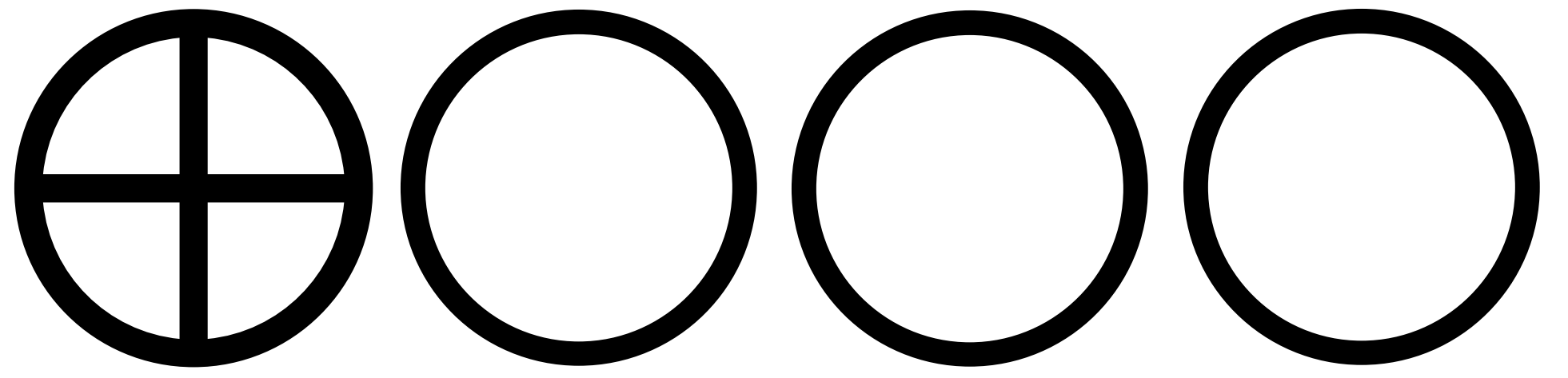 |
| Iglesias-Gutiérrez (2008) | * | Yes |  | 1 | 1 | 1 | 1 | 0 | 1 | 0 | 0 | 1 | 1 | 70% |  | 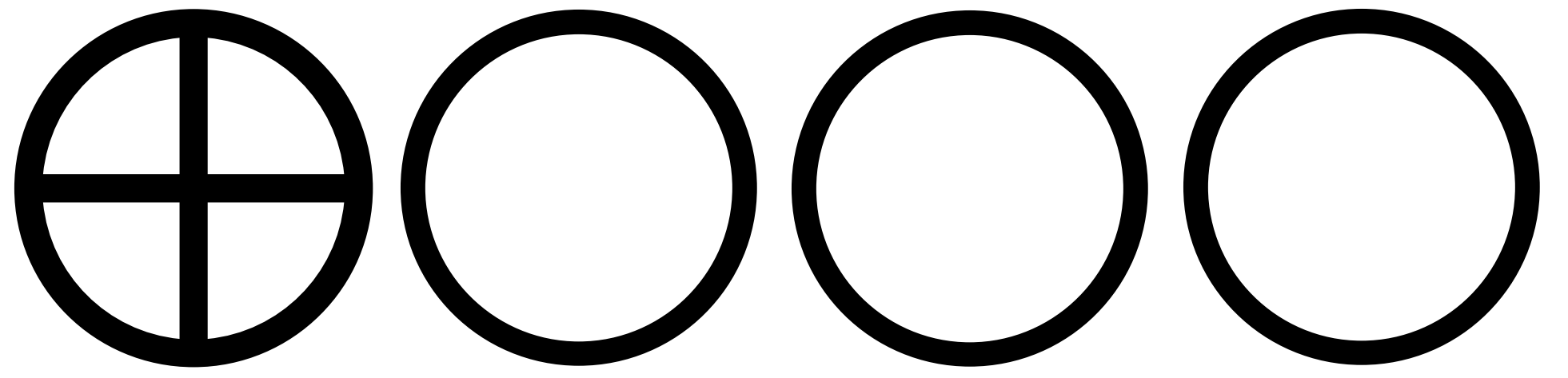 |
| Kabasakalis (2007) | * | Yes |  | 1 | 1 | 1 | 1 | 1 | 1 | 0 | 0 | 1 | 1 | 80% |  | 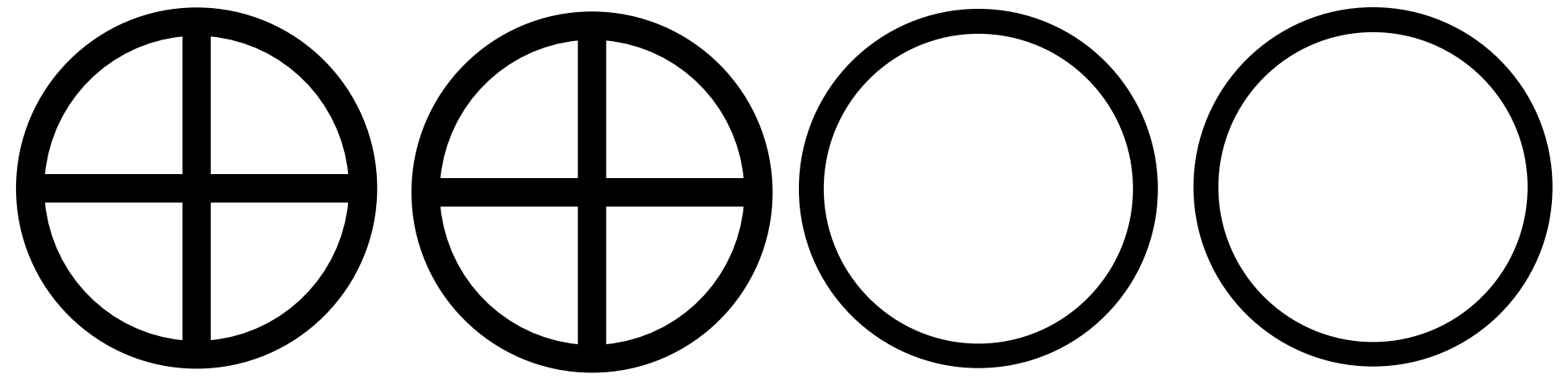 |
| Leblanc (2002) | * | * |  | 1 | 1 | 1 | 1 | 1 | 0 | 0 | 0 | 1 | 1 | 70% |  | 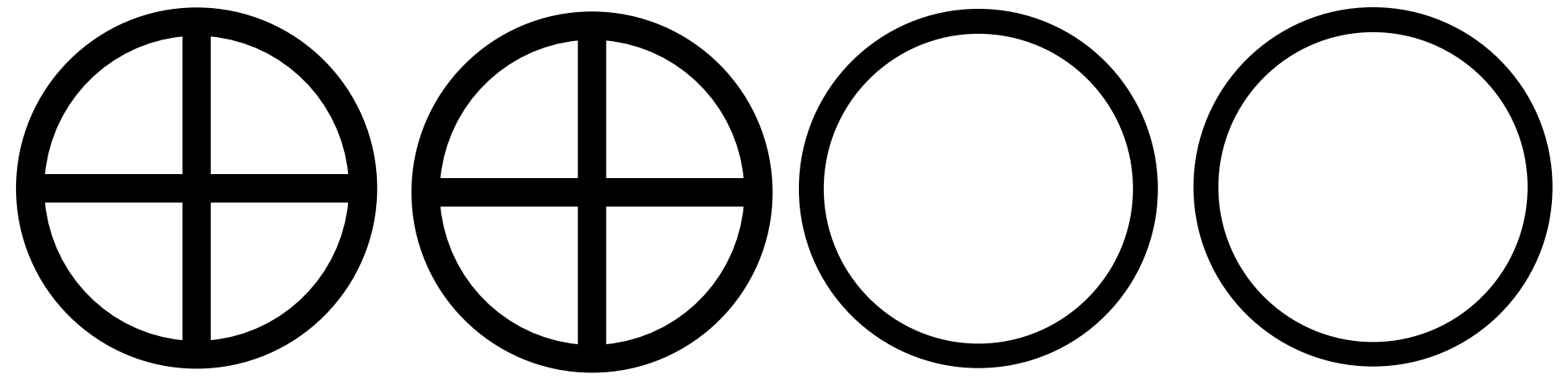 |
| Martínez (2011) | * | Yes |  | 1 | 1 | 1 | 1 | 1 | 0 | 0 | 0 | 1 | 1 | 70% |  | 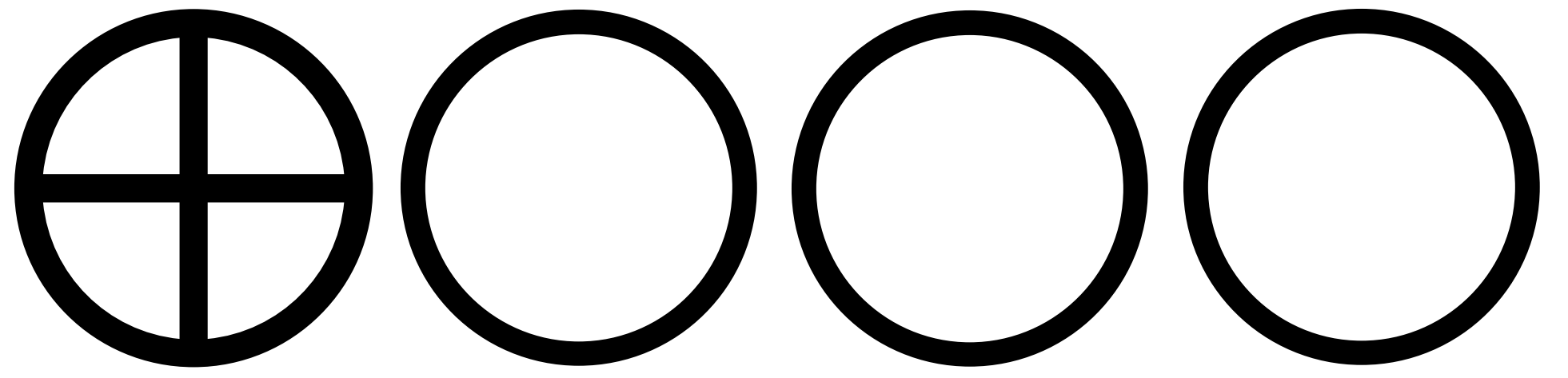 |
| Meckel (2008) | * | Yes |  | 1 | 1 | 1 | 1 | 1 | 0 | 0 | 0 | 1 | 1 | 70% |  | 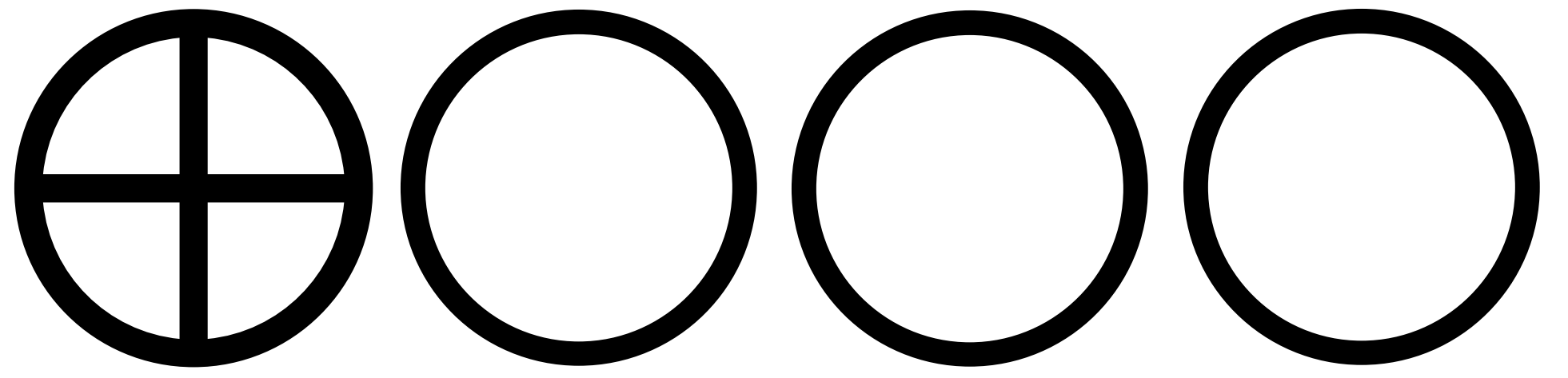 |
| Papadopoulou (2002) | * | * |  | 1 | 1 | 1 | 1 | 1 | 0 | 0 | 0 | 1 | 1 | 70% |  | 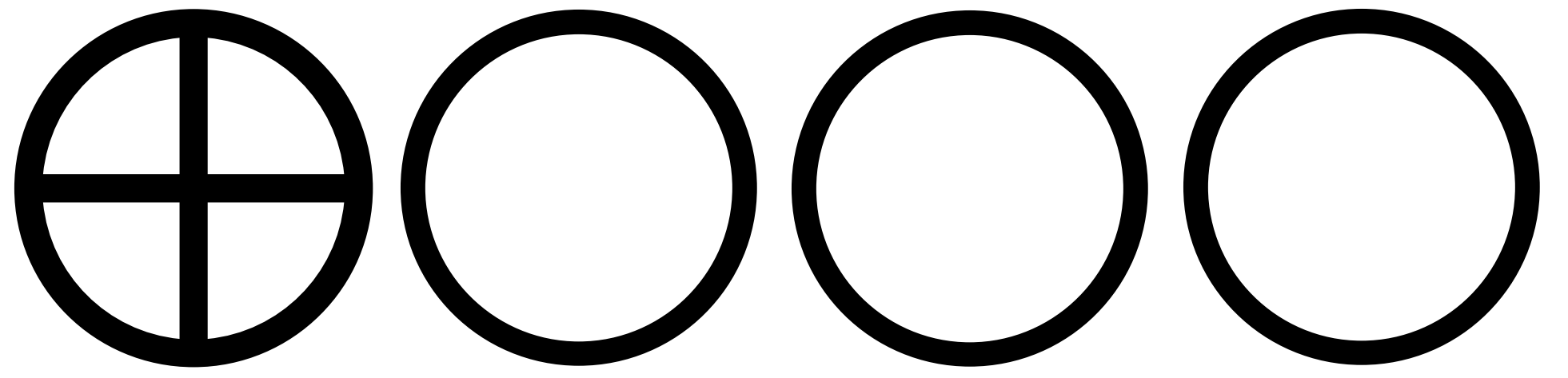 |
| Ruiz (2005) | * | Yes |  | 1 | 1 | 1 | 1 | 1 | 1 | 0 | 0 | 1 | 1 | 80% |  | 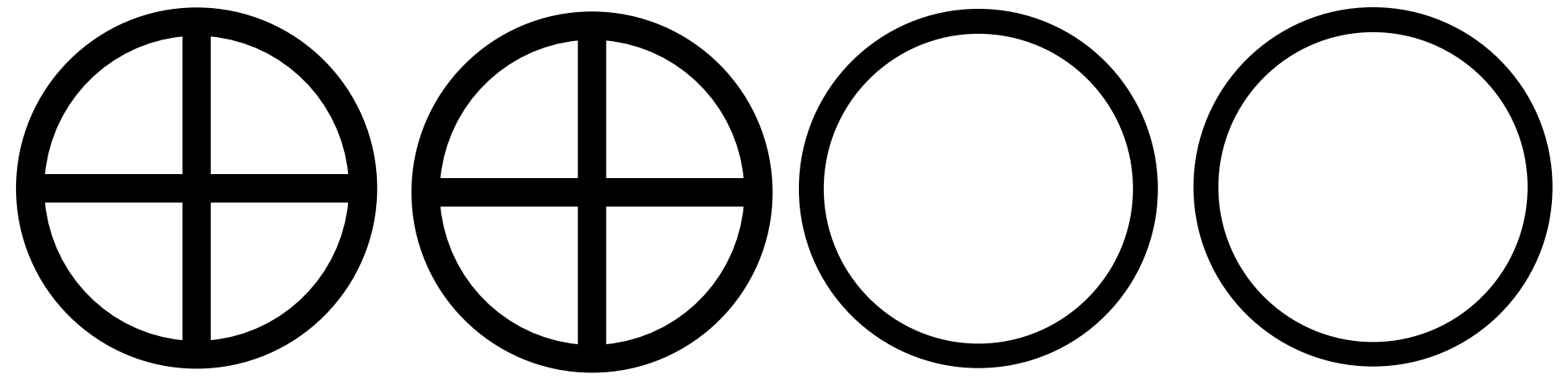 |
| Sousa (2008) | No | Yes |  | 1 | 1 | 1 | 1 | 1 | 1 | 1 | 1 | 1 | 1 | 100% |  | 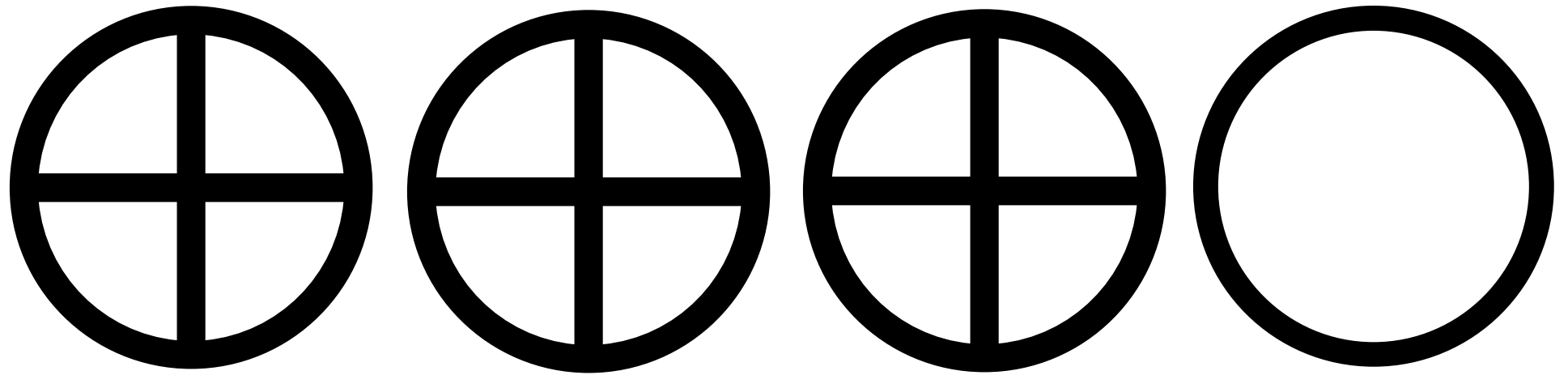 |
| Thivel (2015) | No | * |  | 1 | 1 | 1 | 1 | 1 | 1 | 0 | 0 | 1 | 1 | 80% |  | 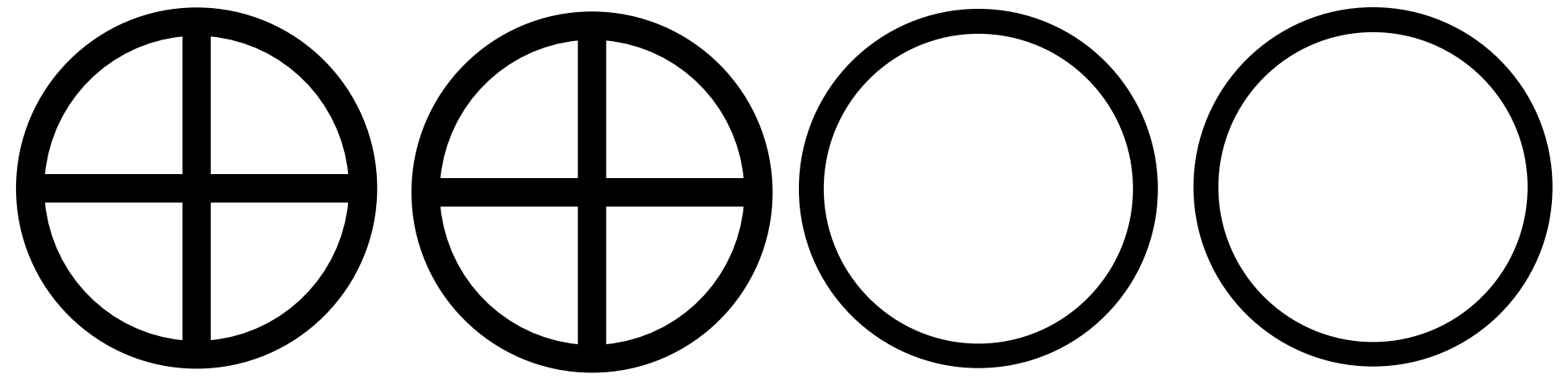 |
| Tong (2012) | * | Yes |  | 1 | 1 | 1 | 1 | 1 | 0 | 0 | 0 | 1 | 1 | 70% |  | 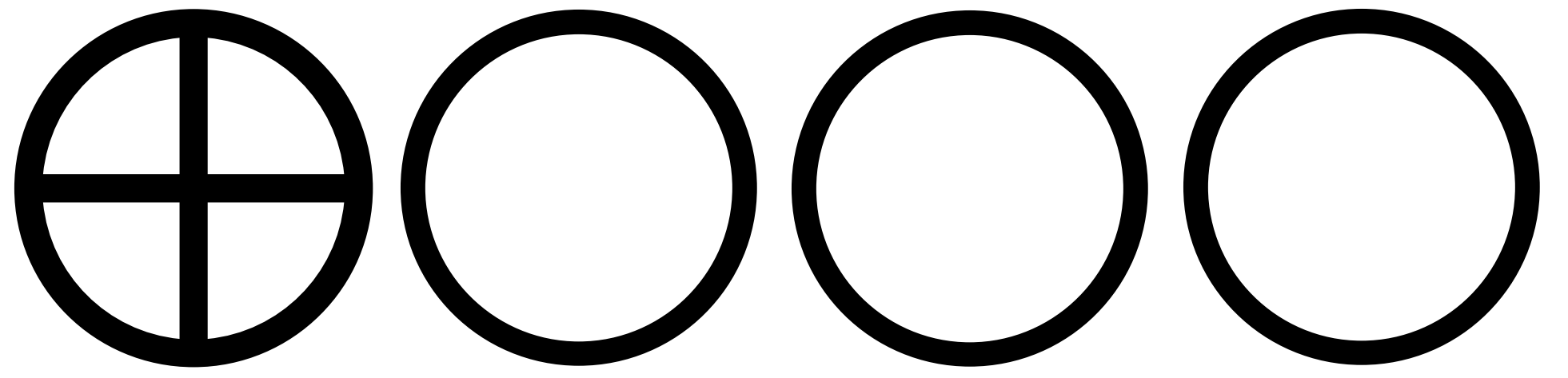 |
| Ziegler (1998) | * | Yes |  | 1 | 1 | 1 | 1 | 1 | 0 | 0 | 0 | 1 | 1 | 70% |  | 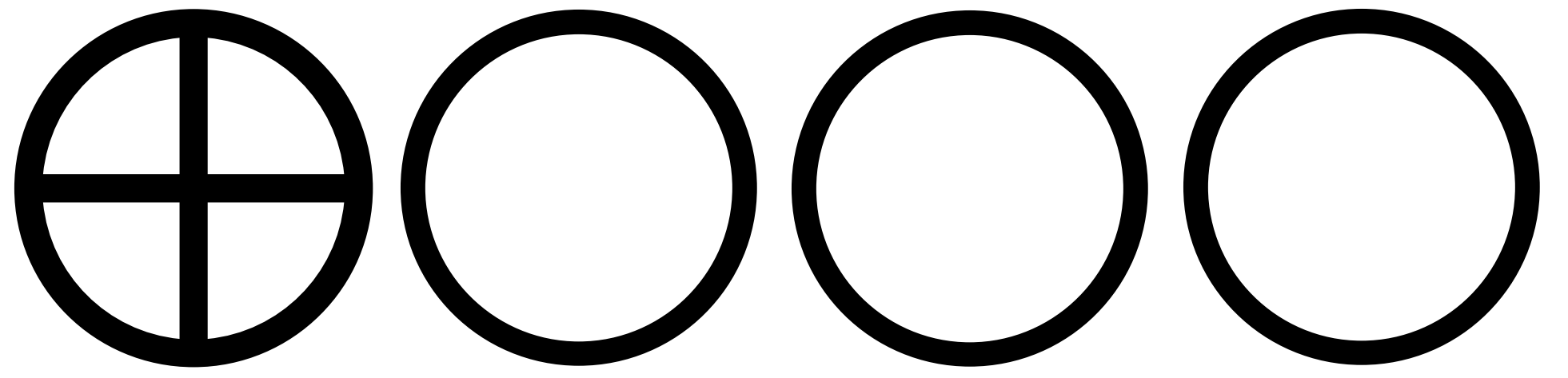 |
| Ziegler (2002) | * | Yes |  | 1 | 1 | 0 | 1 | 1 | 0 | 0 | 0 | 1 | 1 | 60% |  | 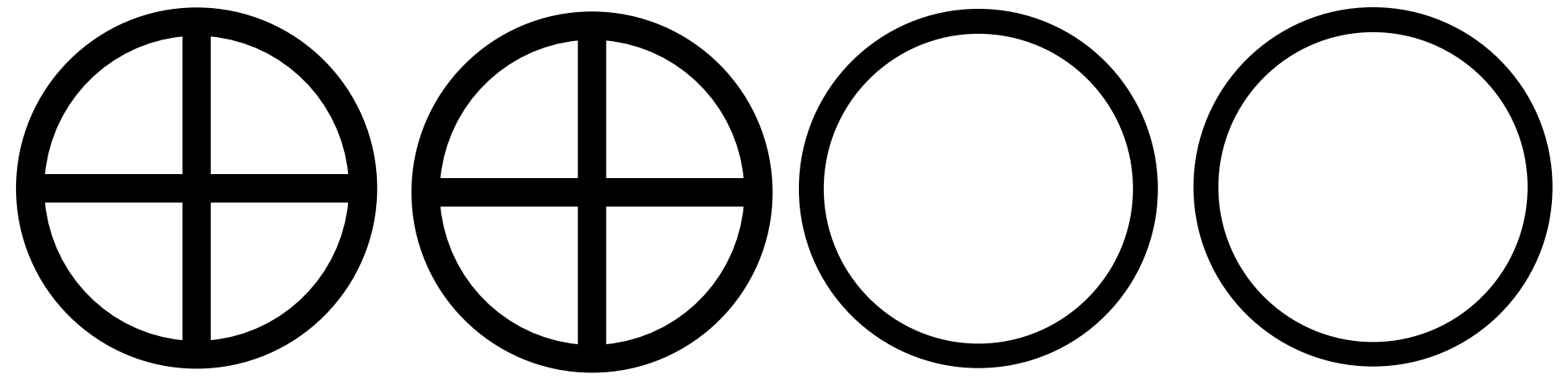 |
| Ziegler (2002) | * | Yes |  | 1 | 1 | 0 | 1 | 1 | 0 | 0 | 0 | 1 | 1 | 60% |  | 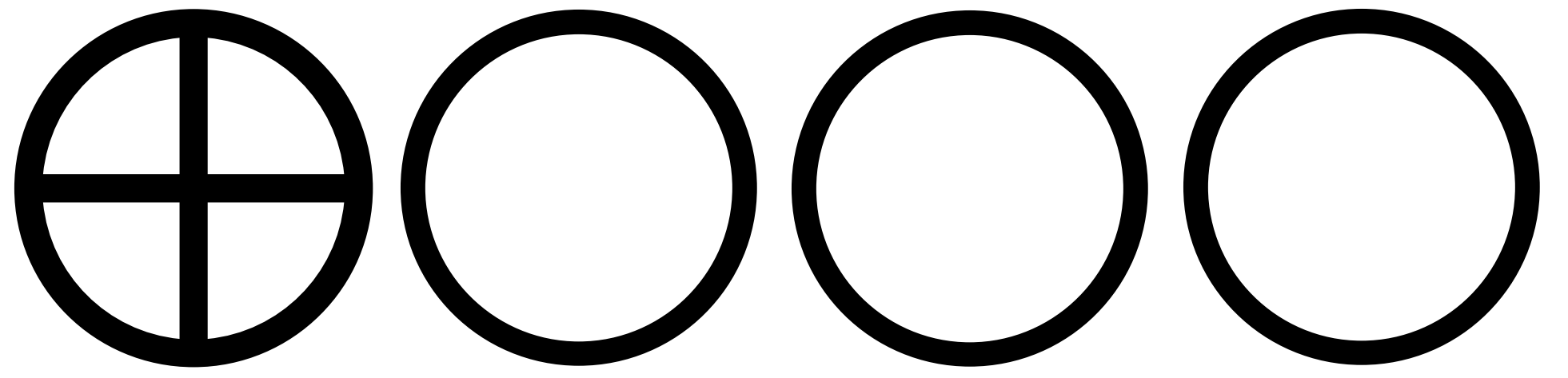 |

Downs and Black checklist: 1, objective clearly stated; 2, main outcomes clearly described to the patient; 3, patient characteristics clearly defined; 4, main findings clearly defined; 5, random variability in estimates provided; 6, probability values reported; 7, sample target representative of population; 8, sample recruitment representative of population; 9, statistical tests used appropriately; and 10, primary outcomes valid/reliable. GRADE: Grading of Recommendations, Assessment, Development and Evaluations. One filled circle: very low quality; two filled circles: low quality; three filled circles: moderate quality; four filled circles: high quality. * Not mentioned.
